# Mutated *RAP1GDS1* causes a new syndrome of dysmorphic feature, intellectual disability & speech delay

**DOI:** 10.1002/acn3.51059

**Published:** 2020-05-19

**Authors:** Abdulaziz Asiri, Essra Aloyouni, Muhammad Umair, Yusra Alyafee, Abeer Al Tuwaijri, Kheloud M. Alhamoudi, Bader Almuzzaini, Abeer Al Baz, Deemah Alwadaani, Marwan Nashabat, Majid Alfadhel

**Affiliations:** ^1^ Medical Genomics Research Department King Abdullah International Medical Research Center (KAIMRC) King Saud Bin Abdulaziz University for Health Sciences King AbdulAziz Medical City Ministry of National Guard Health Affairs Riyadh Kingdom of Saudi Arabia; ^2^ Division of Genetics Department of Pediatrics King Abdullah Specialized Children’s Hospital King Saud Bin Abdulaziz University for Health Sciences King Abdulaziz Medical City Riyadh Saudi Arabia

## Abstract

**Background:**

RAP1GDS1 (RAP1, GTP‐GDP dissociation stimulator 1), also known as SmgGDS, is a guanine nucleotide exchange factor (GEF) that regulates small GTPases, including, RHOA, RAC1, and KRAS. RAP1GDS1 was shown to be highly expressed in different tissue types including the brain. However, mutations in the *RAP1GDS1* gene associated with human diseases have not previously been reported.

**Methods:**

We report on four affected individuals, presenting intellectual disability, global developmental delay (GDD), and hypotonia. The probands’ DNA was subjected to whole‐genome sequencing, revealing a homozygous splice acceptor site mutation in the *RAP1GDS1* gene (1444‐1G > A). Sanger sequencing was performed to confirm the segregation of the variant in two Saudi families. The possible aberrant splicing in the patients’ RNA was investigated using RT‐PCR and changes in mRNA expression of the patients were confirmed using qRT‐PCR.

**Results:**

The identified splice variant was found to segregate within the two families. RT‐PCR showed that the mutation affected *RAP1GDS1* gene splicing, resulting in the production of aberrant transcripts in the affected individuals. Quantitative gene expression analysis demonstrated that the *RAP1GDS1* mRNA expression in all the probands was significantly decreased compared to that of the control, and Sanger sequencing of the probands’ cDNA revealed skipping of exon 13, further strengthening the pathogenicity of this variant.

**Conclusion:**

We are the first to report the mutation of the *RAP1GDS1* gene as a potential cause of GDD and hypotonia. However, further investigations into the molecular mechanisms involved are required to confirm the role of *RAP1GDS1* gene in causing GDD and hypotonia.

## Introduction

Global developmental delay (GDD) is defined as a failure in achieving two or more specific developmental domains, including gross or fine motor skills, cognition, speech and language, and day‐to‐day social/personal activities, in children under the age of five. The prevalence of GDD is uncertain and disputed; however, estimates of the affected children range between 1% and 3%.^1^

RAP1GDS1 (RAP1, GTP‐GDP dissociation stimulator (1), also known as SmgGDS, is an atypical guanine nucleotide exchange factor (GEF) that is involved in the regulation of both cell migration and proliferation, and is overexpressed in many cancer types, including non‐small cell lung carcinoma,^2^, ^3^ breast cancer,^3^ prostate cancer,^4^ and pancreatic cancer.^3^ In addition to its general role as a GEF, RAP1GDS1 plays a critical role in the regulation of small GTPases, including those involved in trafficking, localization, and molecular chaperone functions. Several studies have shown that RAP1GDS1 promotes the binding of GTP through a number of GTPases, including Ras‐related C3 botulinum toxin substrate 1 (Rac1),^5^, ^6^ Ras homologous member A (RhoA),^7^, ^8^ Rap1A and Rap1B,^8^, ^9^ and K‐Ras4B.^8^ RAP1GDS1 regulates the synthesis of DNA induced by Rap1A^10^ and promotes K‐ras4B transformation activity in many cell types.^11^ *RAP1GDS1* consists of two main splice variants, SmgGDS‐558 (NCBI accession # NP_001093899) and SmgGDS‐607 (NCBI accession # NP_001093897), which are reported to have different physiological roles in posttranslational modification of RhoA.^12^, ^13^ However, the understanding of biological functions of the *RAP1GDS1* gene and the potential mechanisms involved are sparse.

In this study, we used whole genome sequencing (WGS) to assess the genetic cause of GDD and hypotonia in two consanguineous Saudi families. Sanger sequencing followed by RT‐qPCR was performed to validate pathogenicity of the novel homozygous splice site mutation in the *RAP1GDS1* gene. To the best of our knowledge, this study is the first to report that a mutation in the *RAP1GDS1* gene is a potential cause of GDD and hypotonia.

## Materials and Methods

### Study approval

This study was approved by the Institutional Review Board of King Abdullah International Medical Research Center (KAIMRC), Riyadh, Saudi Arabia. The patients went through a full clinical assessment for neurological phenotypes at the Genetics clinic of King Abdulaziz Medical City in Riyadh, Saudi Arabia. The standard consent to perform clinical genomic studies was obtained for WGS of the patients and family members. Written informed consent for conducting the study and for publication of clinical data, and photographs were signed by the parents.

### Genomic DNA extraction

Peripheral blood samples were collected from 23 individuals in tubes containing EDTA. Genomic DNA was extracted from whole blood using a QIAamp Blood Midi Kit (Qiagen). The extracted DNA was then quantified using a Nanodrop‐1000 spectrophotometer.

### WGS

WGS was performed at the CENTOGENE laboratory (Rostock, Germany: https://www.centogene.com). 14 Briefly, genomic DNA was fragmented by sonication and Illumina adapters were ligated to generated fragments for subsequent sequencing on the HiSeqX platform (Illumina) to yield an average coverage depth of ~30×. An in‐house bioinformatics pipeline was applied, including base pair calling, alignment of reads to genome assembly GRCh37/hg19, filtering out low quality reads and possible artifacts, and annotation of variants. All disease‐causing variants reported in HGMD,^15^ ClinVar,^16^ and CentoMD^14^ were considered. Furthermore, all variants in gnomAD database^17^ with a minor allele frequency of less than 1% were considered. Evaluation of the identified variants was focused on coding exons and their flanking ±20 intronic bases, taking into account multiple inheritance patterns. Additionally, the provided clinical information and family history of patients were considered for evaluating the identified variants. All variants related to phenotype of the patients, except benign or likely benign variants, were reported.

### Bioinformatics analysis

The potential effect on splicing was predicted using four prediction programs: NNSplice (Berkeley, CA), MutPred Splice (v1.3.2), SKIPPY, Varsome and Human Splice Finder (v2.4.1). The identified variant was not found in different public databases, such as GnomAD (https://gnomad.broadinstitute.org), Exome Variant Server (http://evs.gs.washington.edu/EVS/), 1000 Genomes (http://www.1000genomes.org), Exome Aggregation Consortium ExAC (http://exac.broadinstitute.org/), and dbSNP (http://www.ncbi.nlm.nih.gov/SNP/).

### Mutation validation and co‐segregation analysis

Sanger sequencing was applied to confirm the identified variants. The *RAP1GDS1* mutation (c.1444‐1G > A) was amplified and sequenced through standard procedures using the primers: forward 5′‐CGCAAAACCACTCAATG‐3′ and reverse 5′‐GCCAAAGCAACAAGAGCTTC‐3′, with an annealing temperature of 60°C and 40 amplification cycles. The segregation analysis was performed in all the members of the two families.

### Splicing site mutation analysis

To check the effect of the identified variant on splice site of *RAP1GDS1* gene, RNA was extracted from fresh blood samples of four patients homozygous for *RAP1GDS1* c.1444‐1G > A (two affected from first family and two affected from the second family) and 19 healthy control from two different families, using a standard TRIzol RNA isolation protocol (Invitrogen). RNA thus obtained was then reverse‐transcribed using a high‐capacity cDNA reverse transcription kit (Applied Biosystems). Real time‐PCR was performed with 1 μL cDNA per 15 μL reaction using the following primers: forward 5′‐CTGCCCTTATACGACACAGTA‐3′ and reverse 5′‐GATTTCAGGAGCACTTCTCTC–3′ with an annealing temperature of 57°C and 40 amplification cycles. The PCR products were then run on 2% agarose gel electrophoresis set‐up supplemented with SYBR™ Safe Stain (Invitrogen) and subsequently visualized on a UV transilluminator. The effect of the splice mutation identified in both the affected brothers was assessed by amplifying exons 12–14 in the *RAP1GDS1* gene by RT‐PCR using the same primer pairs mentioned above and analyzing the obtained PCR products by Sanger sequencing.

To quantify the gene expression of *RAP1GDS1* in all family members, quantitative real time PCR (qPCR) was performed on the QuantStudio 6 Flex Real‐Time PCR System (Applied Biosystems) using SYBR^®^ Green PCR Master Mix (Applied Biosystems) using the following primers: forward 5′‐ TGA AGC CAA AGA TCA TGC TGG‐3′ and reverse 5′‐ ATG CTT GAT GCC ACC ACT CTG‐3′ under PCR cycle conditions of 95°C for 10 min, followed by 40× (95°C for 15 sec, annealing at 57°C for 30 sec). No template control (NTC) was used as a negative control for each experiment. Each sample was run in triplicate. GAPDH (Glyceraldehyde‐3‐Phosphate Dehydrogenase) was used as the endogenous control and the 2‐ΔΔCt method was used for gene quantification since the primer pairs had similar efficiency.

### Statistical analysis

All statistical analysis was performed using GraphPad Prism (version 8.1). Results were tested for a normal distribution, and analysis of variance (one‐way ANOVA) was applied. Differences in the means were considered statistically significant if *P* < 0.05.

## Results

### Clinical evaluation

#### Family 1

Patient IV‐9 is a 4‐year‐old boy. He was born to consanguineous Saudi parents. The prenatal ultrasound showed a horseshoe kidney; otherwise, the pregnancy course and delivery were normal. All the parameters at birth were within the normal range—length: 50.5 cm, weight: 3550 gm, and head circumference: 34.5 cm. At the age of 8 months, he was observed to have a gross motor delay. At that time, he was only able to roll over in the bed but was unable to sit even with support. His growth parameters were appropriate for his age. A neurology team was involved in his treatment, and all the initial investigations requested for the patient, including complete metabolic workup and brain MRI, were normal.

At the age of 2 years, the patient was referred to a genetics clinic for developmental delay. He was able to walk only with support and to say a few single words. The family history was remarkable for one sibling with a similar presentation.

On examination, the patient's growth parameters were appropriate for his age. He had dysmorphic features, such as arched eyebrows, bulbous nose, flat philtrum, thin lips, retrognathia, and right ear tags. The patient also exhibited axial hypotonia and hyporeflexia. Other systemic examinations were normal.

Currently, the patient is 4‐years old and still has a mild axial hypotonia, presenting an unsteady gait while running. The patient has shown a delay in expressive speech; he can only combine two words phrases. He can understand and obey complex commands. Recently, the patient has started displaying aggressive behavior and self‐mutilation, mainly toward his hands. His latest growth parameters were as follows—height: 102 cm (50th percentile), weight: 16.2 kg (50th percentile), and head circumference 49 cm (10th–25th percentile). On further examination, his hypotonia has improved relatively, and joint laxity is noticed in addition to bite marks on the hands.

Patient IV‐10 is a younger sibling of patient IV‐9. The prenatal course was normal and the delivery was at 37 weeks plus 4 days by spontaneous vaginal delivery. All his parameters at birth were within the normal range—length: 50 cm, weight: 3220 gm, and head circumference: 34.5 cm. The patient was stable and was discharged after 2 days of phototherapy.

At the age of 9 months, he was admitted to the hospital due to febrile illness and diarrhea due to hepatitis A infection. During the admission, the patient was noted to have a gross motor delay and was unable to sit without support; however, he was interacting well with the surroundings. The initial metabolic investigations were normal.

As this patient also had the same phenotype as his sibling, he was also referred to the genetics clinic. The patient had a mild axial hypotonia and similar facial features as his brother’s, such as arched eyebrows, bulbous nose, flat philtrum, and thin lips. He had hyporeflexia as well.

Currently, the patient is 40 months old with relatively improved hypotonia. He can reach for objects and feed himself. The patient can obey complex commands; however, he can only utter single words. His latest growth parameters were as follows, height: 98 cm (25th–50th percentile), weight: 13.8 kg (50th percentile), and head circumference 49 cm (25th percentile).

#### Family 2

Patient IV‐4 is a 27‐year‐old male. He was born to consanguineous Saudi parents with an unremarkable pregnancy course and delivery. The patient's growth parameters after delivery were within the normal range. After delivery, he was found to have bilateral club feet and was treated using conventional methods. At the age of 6 months, he was found to have a gross motor delay. He started to walk at the age of 4 years; however, he was unable to combine two words until school‐age but could understand commands.

At the age of 10 years, he developed generalized tonic‐clonic seizures and was started on valproic acid treatment, leading to a partial control of symptoms. The last seizure was 3 months back. The patient was diagnosed to have an intellectual disability and attended learning‐disability classes. On examination, all the growth parameters were below the third percentile—height: 159.5 cm, weight: 46.4 kg, and head circumference 54 cm.

The patient has dysmorphic facial features, such as a triangular face, bulbous nose, retrognathia, low‐set ears, and short philtrum. The elbow joints are externally rotated and there is a mild inward rotation of the feet. The patient has mild axial hypotonia and dorsal clonus. Currently, he exhibits a speech delay; however, he understands some basic commands and conversations. All other systemic molecular and metabolic investigations are normal.

Patient IV‐2 is a 5‐year‐old boy. He is the stepbrother of patient IV‐4 and his parents are first cousins. The pregnancy course and delivery were unremarkable. The patient's growth parameters after delivery were within the normal range. Since birth, the patient was found to have bilateral club feet and were floppy. Additionally, he has a global developmental delay.

Currently, his height and weight are below the third percentile—height: 97 cm, weight: 13.7 kg, and head circumference: 50 cm at the 25th percentile. The patient exhibits dysmorphic features, that is, a triangular face, bulbous nose, everted ears, short and flat philtrum, and retrognathia. The patient displays mild axial hypotonia and he has club feet showing dorsal clonus. All other systemic examinations were normal.

#### Investigations

The biochemical and genetic investigations, such as acylcarnitine profile; ammonia, lactic acid, and creatine kinase (CK) levels; very long chain fatty acid (VLCFA); total homocysteine; urinary amino acids, organic acids, and purine and pyrimidines; urinary creatine and guanidinoacetate; liver enzymes; coagulation profiles; lipid profile; karyotype; array comparative genomic hybridization (CGH); and whole exome sequencing (WES) were found to be normal in all the patients.

### WGS identified a novel variant in *RAP1GDS1* gene

**Figure 1:**
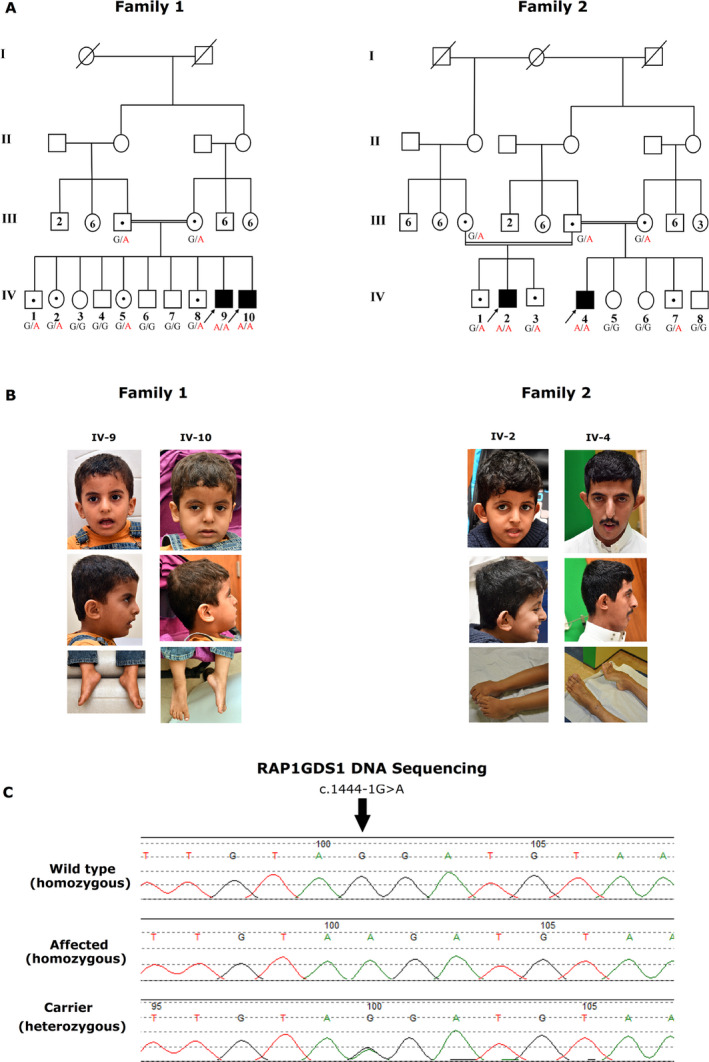
Diagrammatic representation of Clinical features, pedigree, and sequence chromatograms of the identified pathogenic variant in the *RAP1GDS1* gene. (A) Pedigree of the analyzed family members. The allelic status is given below each tested individual. Symbols are as follows: filled, affected; empty, unaffected; dotted, heterozygous carrier; black arrow, individuals subjected to whole genome sequencing (WGS). (B) Photographs showing the phenotypes of the patient. The pictures show the facial dysmorphic, global developmental delay, and hypotonia features. Written informed consent was obtained from the patient’s parents for the publication of images. (C) Segregation of the identified splice site variant (c.1444‐1G > A) in both families.

WGS was performed on the DNA of all the affected individuals (Fig. 1B) using HiSeqX platform (Illumina, Cenogene, Germany) with an average coverage depth of ~30×. Data analysis revealed the presence of a single homozygous splice acceptor site (c.1444‐1G > A) in the *RAP1GDS1* gene (NM_00100426.2).

### Sanger sequencing

The identified splice variant was found to segregate with the disease phenotype as confirmed by Sanger sequencing (Fig. 1C). The variant (c.1444‐1G > A), in a homozygous state, is not reported in any of the available public databases, including dbSNP, 1000 Genomes, internal database (in‐house), and ExAC. Additionally, the variant was checked in 2000 in‐house exomes from unrelated Saudi Arabian individuals, to exclude the possibility of polymorphic nature of the variant (Table 1). The identified splice site variant, c.1444‐1G > A, was predicted to be pathogenic using different *in silico* analyses tools as mentioned previously. These tools revealed that the identified variant completely abolished the corresponding acceptor splice site, as expected for DNA changes involving −1 and −2 bases of intron acceptor sequences.

**Table 1:** Characterization of splice site variant (c.1444‐1G> A) identified in the present family.

Chr. position (hg19)	Chr4:99355086
Reference allele	G
Alternate allele	A
Gene	*RAP1GDS1*
MIM	179502
Gene bank	NM_001100426.2
cDNA Change	c.1444‐1G > A
Amino acid change	–
Variant type	Splice site SNV^1^
Variant status	Novel
ESP	–
ExAC_Freq	–
genomAD	–
dbSNP	–
ClinVar_status	–
SIFT Score &prediction	–
Polyphen2 score & prediction	–
dbscSNV	0.9999
Mutation taster score &prediction	–
FATHMM_SCORE & prediction	–
CADD score	–
ACMG classification	PVS1^1^
Other information's	Homozygous

^1^ SNV, single nucleotide variant; PVS1, pathogenic very strong 1.

### Characterization of the splice site variant

**Figure 2:**
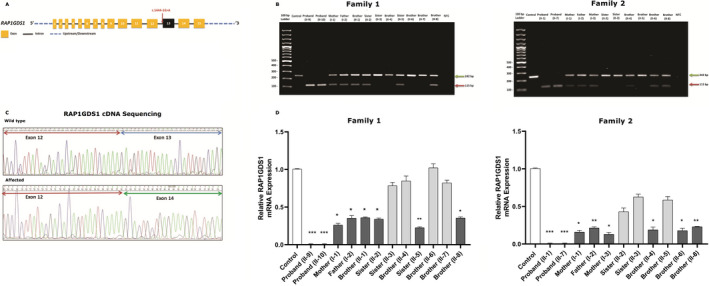
Molecular characterization of c.1444‐1G > A splicing variant in *RAP1GDS1* gene. (A) Schematic representation of the *RAP1GDS1* genomic region: exons (Yellow), intron (Black), UTRs (Blue). Red arrow represents the identified splice acceptor site variant. (B) Agarose gel (2%) demonstrating the results of the RT‐PCR performed on the RNA extracted from the peripheral blood mononuclear cells of the unrelated normal control and the family members. PCR amplification of the region around the splice site variant showed a decrease in the size of the *RAP1GDS1* gene product in all probands from families 1 and 2 (Red arrow, 115 bp) following the skipping of exon 13 compared the control sample (Green arrow, 242 bp). The carrier individuals of the same mutation (heterozygous) from both families showed two bands. No template control (NTC). (C) Representing Sanger sequencing of the wild‐type control and affected individual’s cDNA. Chromatograms sequence analysis showing a complete skipping of exon 13 (blue arrow) in the affected individuals compared to the controls. (D) Quantitative real time‐ PCR showed that the *RAP1GDS1* mRNA expression level was significantly inhibited in the proband IV‐9 and IV‐10 from family 1 and proband IV‐4 and IV‐2 from family 2 as compared to the normal control. There was also a significant decrease in the mRNA level of *RAP1GDS1* expression in all carrier members from both families compared to the wild type control, as they are heterozygous for the mutation. Results are represented as the mean ± SD of three independent experiments (one‐way *ANOVA*). *P < 0.05, **P < 0.01, ***P < 0.001.

**Figure 3:**
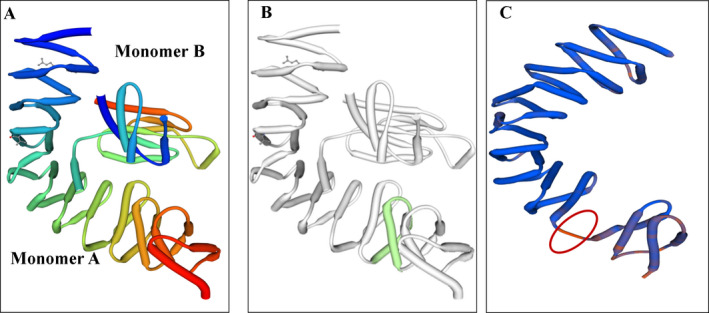
Crystal shape protein model of the RAP1GDS1. (A) Model of the wild type RAP1GDS1 structure consisting monomer A and B. (B) Model of RAP1GDS1 protein with the location of the effected armadillo motif highlighted in green (from Asp 361 to Leu 402). (C) Expected protein model for the mutated RAP1GDS1 (c.1444‐1G > A) showed complete skipping for the armadillo motif (circled in red) with significant change in the protein structure. The images were created by SWISS‐MODEL.

To gain a better understanding of the effect of this single variant, the possible aberrant splicing of the RNA obtained from the patients was confirmed by RT‐PCR. In addition, we examined all members of both the families and unrelated normal control samples. Agarose gel electrophoresis of the obtained RT‐PCR products demonstrated that this variant affected *RAP1GDS1* gene splicing, thereby leading to a reduction in the size of the gene (Fig. 2B). This finding was further validated by sequencing the cDNA of all the probands from the two families (Fig. 2C). To explain in details, the c.1444‐1G > A variant mainly causes a complete skipping of exon 13 that may result in a shorter alternative *RAP1GDS1* transcript (Fig. 2). This alternative transcript, characterized by a complete deletion of one conserved armadillo motif (Fig. 3C), results in a change of the size and structure of the protein, which ultimately may lead to a shorter non‐functional protein. Furthermore, we quantified the mRNA levels of the *RAP1GDS1* gene by qRT‐PCR of all members of the two families, in addition to unrelated normal control samples (Fig. 2D). The results confirmed that the patients carrying the homozygous c.1444‐1G > A variant showed a complete reduction in the mRNA expression levels of *RAP1GDS1* following the skipping of exon 13 compared to that in normal controls, further strengthening the pathogenicity of the identified splicing variant (Table 2).

**Table 2:** Clinical description of subjects with Biallelic *RAP1GDS1* mutation.

Parameters	Family 1		Family 2	
***RAP1GDS1* variant** NM_001100426.2	IV‐9	IV‐10	IV‐4	IV‐2
Ethnicity	Arab‐Saudi	Arab‐Saudi	Arab‐Saudi	Arab‐Saudi
Consanguinity	+	+	+	+
Perinatal history	−	−	−	−
Pregnancy course	Normal	Normal	Normal	Normal
Delivery	Normal	Normal	Normal	Normal
APGAR	Normal	Normal	Normal	Normal
Postnatal growth parameters	Normal	Normal	Normal	Normal
Clinical features				
Dysmorphic	+	+	+	+
Developmental delay				
Motor	+	+	+	+
Receptive Speech	−	−	−	−
Expressive speech	+	+	+	+
Seizures	−	−	+	−
Club feet	−	−	+	+
Horseshoe kidney	+	−	−	−
Axial Hypotonia	+	+	+	+
Latest growth parameters	Normal	Normal	<5th percentile	<5th percentile
Intellectual disability	+	+	+	+

However, we were unable to perform further experiments, such as protein quantification to study the functional activity of this novel gene, due to refusal by the affected members to provide additional blood samples and skin biopsies.

## Discussion

Global developmental delay (GDD) and intellectual disability (ID) affect a large number of individuals, worldwide, and many genes underlying GDD/ID have been identified; however, a large fraction of such complex brain‐related disorders remain unexplained.^18^, ^19^, ^20^ Some ID/GDD cases may result due to environmental factors, and it is more likely that these patients harbor pathogenic, highly penetrant variations in novel disease‐associated genes.^21^

Rho GTPases represent a subgroup of the Ras superfamily of small GTP‐binding proteins and consist of more than 20 members in mammals. The most widely investigated members of the Rho family in the nervous system are the Ras‐related C3 botulinum toxin substrate 1 (Rac1), Ras homologous member A (RhoA), and the cell division cycle 42 (CDC42).^22^

Similar to other GTPase members of the Ras superfamily, the majority of Rho GTPases cycle between GTP‐ (active) and GDP‐ (inactive) bound states. The GDP/GTP cycle is regulated by protein‐protein interactions through either the activation of guanine nucleotide exchange factors (GEF) or inhibition of the GTPase activating protein (GAP), resulting in downstream targets activation.^22^, ^23^ In the adult brain, small GTPases regulate biological events of the actin cytoskeleton of the excitatory synapse, thus contributing to synaptic plasticity.^23^, ^24^

Mutations in different genes encoding the Rho GTPases has been associated with ID and GDD phenotypes in literature.^25^ These include homozygous loss‐of‐function variants in the *ARHGEF2* gene (OMIM 607560), associated with mental retardations in humans, resulting in the inhibition of the RhoA‐ROCK‐MLC pathway activation, which in turn is critical for cell motility; altered migration of pre‐cerebellar immature neurons has been observed in the *ARHGEF2*−/− mice.^26^ Additionally, variants in the *RAC1* gene (OMIM 602048) have been observed in patients with neuro‐developmental diseases, such as ID, which present in several phenotypic spectra, including microcephaly, cerebellar abnormalities, or macrocephaly.^27^ Similarly, the *ARHGAP15* gene (OMIM 610578) encodes for a brain‐specific and Rac1‐specific GAP that plays a key role in the reduction of the GTP‐bound levels of intracellular Rac1 in the brain. A loss of *ARHGAP15* gene function has been reported in patients with a rare genetic mutation in Mowat‐Wilson disease, which is characterized by several phenotypes, including autism and speech impairment, and severe neurological and cognitive deficits.^28^

In light of the data presented above, the affected individuals in our study, exhibiting features, such as intellectual disability and speech delay were subjected to WGS using standard methods. WGS filtering steps revealed a novel splice acceptor site variant (1444‐1G > A) in intron 12 of the *RAP1GDS1* gene located on chromosome 4q23. This splice site variant was considered pathogenic employing different online available splice analysis tools. The RT‐PCR and Sanger sequencing analyses followed by qRT‐PCR revealed a significant decrease in the expression of *RAP1GDS1* gene in all the probands compared to that in the control samples, thus suggesting skipping of the exon 13 (Fig. 2). The identified variant may alter the splicing pattern of the *RAP1GDS1* gene and may affect pre‐mRNA splicing by altering the acceptor splice site at −1 position and possibly using a cryptic splice site. In addition, the variant, is predicted to result in a truncated RAP1GDS1 protein lacking several key domains involved in the interaction of RAP1GDS1 with different downstream target sites.^29^ It has been reported that the most common consequence of spliced variants is the skipping of one or more exons. However, in certain cases, activation of cryptic 5′ (donor) or 3′ (acceptor) splice site and partial or full retention of specific introns may occur.^30^

On the whole, our results will add to the basic knowledge underlying *RAP1GDS1* pathogenesis. This might raise new questions associated with molecular pathogenesis of mutations affecting small GTPases and might have important implications for clinical genomics. In summary, we have demonstrated that the homozygous splice acceptor site variant in the *RAP1GDS1* leads to ID, GDD, hypotonia, and related phenotypes. The expression studies using RT‐qPCR also showed that this variant might alter the key functions of *RAP1GDS1*. We propose to name this syndrome as “Alfadhel syndrome.” Our observations add to the diverse and pleiotropic group of Mendelian disorders caused by variations in the *RAP1GDS1* gene and related RAC1 and RhoA pathways.^5^, ^7^ However, recruiting more cases and further functional studies are required to gain insights into the molecular mechanisms involved, which might confirm the precise role of *RAP1GDS1* gene in causing ID, GDD, and hypotonia in humans.

## Conflict of Interest

The authors have no competing interests.

## Author Contributions

AA designed the study, analyzed the data, and wrote the manuscript. EA conducted the majority of the experimental work. MU reviewed and edited the manuscript. YA and AA performed work associated with Sanger sequencing and segregation analysis. KMA and BA edited the manuscript. AA and DA assisted with some experimental work. MN participated in the clinical diagnosis and management of patients. MF contributed to the clinical diagnosis and management of patients, and edited the manuscript. All authors have read and approved the final manuscript.

## References

[1] Vasudevan P , Suri M . A clinical approach to developmental delay and intellectual disability. Clin Med 2017;17:558–561. 10.7861/clinmedicine.17-6-558 PMC6297696 29196358

[2] Tew GW , Lorimer EL , Berg TJ , et al. SmgGDS regulates cell proliferation, migration, and NF‐κB transcriptional activity in non‐small cell lung carcinoma. J Biol Chem 2008;283:963–976. 17951244 10.1074/jbc.M707526200

[3] Schuld N , Hauser A , Gastonguay A , et al. SmgGDS‐558 regulates the cell cycle in pancreatic, non‐small cell lung, and breast cancers. Cell Cycle 2014;13:941–952. 24552806 10.4161/cc.27804 PMC3984317

[4] Zhi H , Yang XJ , Kuhnmuench J , et al. SmgGDS is up‐regulated in prostate carcinoma and promotes tumour phenotypes in prostate cancer cells. J Pathol 2009;217:389–397. 18973191 10.1002/path.2456

[5] Chuang T‐H , Xu X , Quilliam L , Bokoch G . SmgGDS stabilizes nucleotide‐bound and‐free forms of the Rac1 GTP‐binding protein and stimulates GTP/GDP exchange through a substituted enzyme mechanism. Biochem J 1994;303:761–767. 7980444 10.1042/bj3030761 PMC1137612

[6] Yaku H , Sasaki T , Takai Y . The Dbl oncogene product as a GDP/GTP exchange protein for the Rho family: its properties in comparison with those of Smg GDS. Biochem Biophys Res Comm 1994;198:811–817. 8297393 10.1006/bbrc.1994.1116

[7] Hutchinson JP , Eccleston JF . Mechanism of nucleotide release from Rho by the GDP dissociation stimulator protein. Biochemistry 2000;39:11348–11359. 10985780 10.1021/bi0007573

[8] Mizuno T , Kaibuchi K , Yamamoto T , et al. A stimulatory GDP/GTP exchange protein for smg p21 is active on the post‐translationally processed form of c‐Ki‐ras p21 and rhoA p21. Proc Natl Acad Sci 1991;88:6442–6446 1907371 10.1073/pnas.88.15.6442 PMC52101

[9] Hata Y , Kaibuchi K , Kawamura S , et al. Enhancement of the actions of smg p21 GDP/GTP exchange protein by the protein kinase A‐catalyzed phosphorylation of smg p21. J Biol Chem 1991;266:6571–6577. 1901063

[10] Yoshida Y , Kawata M , Miura Y , et al. Microinjection of smg/rap1/Krev‐1 p21 into Swiss 3T3 cells induces DNA synthesis and morphological changes. Mol Cell Biol 1992;12:3407–3414. 1321333 10.1128/mcb.12.8.3407-3414.1992 PMC364589

[11] Fujioka H , Kaibuchi K , Kishi K , et al. Transforming and c‐fos promoter/enhancer‐stimulating activities of a stimulatory GDP/GTP exchange protein for small GTP‐binding proteins. J Biol Chem 1992;267:926–930. 1730682

[12] Berg TJ , Gastonguay AJ , Lorimer EL , et al. Splice variants of SmgGDS control small GTPase prenylation and membrane localization. J Biol Chem 2010;285:35255–35266. 20709748 10.1074/jbc.M110.129916 PMC2975149

[13] Schuld NJ , Vervacke JS , Lorimer EL , et al. The chaperone protein SmgGDS interacts with small GTPases entering the prenylation pathway by recognizing the last amino acid in the CAAX motif. J Biol Chem 2014;289:6862–6876. 24415755 10.1074/jbc.M113.527192 PMC3945348

[14] Trujillano D , Bertoli‐Avella AM , Kumar Kandaswamy K , et al. Clinical exome sequencing: results from 2819 samples reflecting 1000 families. Eur J Hum Genet 2017;25:176–182. 27848944 10.1038/ejhg.2016.146 PMC5255946

[15] Stenson PD , Ball EV , Mort M , et al. The Human Gene Mutation Database (HGMD) and its exploitation in the fields of personalized genomics and molecular evolution. Curr Protoc Bioinformatics 2012;39:1–3. 10.1002/0471250953.bi0113s39 22948725

[16] Landrum MJ , Lee JM , Benson M , et al. ClinVar: public archive of interpretations of clinically relevant variants. Nucleic Acids Res 2015;44:D862–D868. 26582918 10.1093/nar/gkv1222 PMC4702865

[17] Lek M , Karczewski KJ , Minikel EV , et al. Analysis of protein‐coding genetic variation in 60,706 humans. Nature 2016;536:285. 27535533 10.1038/nature19057 PMC5018207

[18] Ropers HH . Genetics of intellectual disability. Curr Opin Genet Dev 2008;18:241–250. 18694825 10.1016/j.gde.2008.07.008

[19] Yang Y , Muzny DM , Xia F , et al. Molecular findings among patients referred for clinical whole‐exome sequencing. JAMA 2014;312:1870–1879. 25326635 10.1001/jama.2014.14601 PMC4326249

[20] Taylor JC , Martin HC , Lise S , et al. Factors influencing success of clinical genome sequencing across a broad spectrum of disorders. Nat Genet 2015;47:717. 25985138 10.1038/ng.3304 PMC4601524

[21] Niemi ME , Martin HC , Rice DL , et al. Common genetic variants contribute to risk of rare severe neurodevelopmental disorders. Nature 2018;562:268. 30258228 10.1038/s41586-018-0566-4 PMC6726472

[22] Azzarelli R , Kerloch T , Pacary E . Regulation of cerebral cortex development by Rho GTPases: insights from in vivo studies. Front Cell Neurosci 2015;8:445. 25610373 10.3389/fncel.2014.00445 PMC4285737

[23] Martino A , Ettorre M , Musilli M , et al. Rho GTPase‐dependent plasticity of dendritic spines in the adult brain. Front Cell Neurosci 2013;7:62. 23734098 10.3389/fncel.2013.00062 PMC3661998

[24] Sawada M , Ohno N , Kawaguchi M , et al. PlexinD1 signaling controls morphological changes and migration termination in newborn neurons. EMBO J 2018;37: e97404 10.15252/embj.201797404 29348324 PMC5813262

[25] Zamboni V , Jones R , Umbach A , et al. Rho gtpases in intellectual disability: From genetics to therapeutic opportunities. Int J Mol Sci 2018;19:1821. 10.3390/ijms19061821 PMC6032284 29925821

[26] Ravindran E , Hu H , Yuzwa SA , et al. Homozygous ARHGEF2 mutation causes intellectual disability and midbrain‐hindbrain malformation. PLoS Genet 2017;13:e1006746. 28453519 10.1371/journal.pgen.1006746 PMC5428974

[27] Lelieveld SH , Wiel L , Venselaar H , et al. Spatial clustering of de novo missense mutations identifies candidate neurodevelopmental disorder‐associated genes. Am J Hum Genet 2017;101:478–484. 28867141 10.1016/j.ajhg.2017.08.004 PMC5591029

[28] Mulatinho MV , de Carvalho Serao CL , Scalco F , et al. Severe intellectual disability, omphalocele, hypospadia and high blood pressure associated to a deletion at 2q22.1q22.3: case report. Mol Cytogenet 2012;5:30. 22686481 10.1186/1755-8166-5-30 PMC3407782

[29] Maquat LE . Nonsense‐mediated mRNA decay: splicing, translation and mRNP dynamics. Nat Rev Mol Cell Biol 2004;5:89. 15040442 10.1038/nrm1310

[30] Krawczak M , Reiss J , Cooper DN . The mutational spectrum of single base‐pair substitutions in mRNA splice junctions of human genes: causes and consequences. Hum Genet 1992;90:41–54. 1427786 10.1007/BF00210743

